# One-step printable platform for high-efficiency metasurfaces down to the deep-ultraviolet region

**DOI:** 10.1038/s41377-023-01086-6

**Published:** 2023-03-08

**Authors:** Joohoon Kim, Wonjoong Kim, Dong Kyo Oh, Hyunjung Kang, Hongyoon Kim, Trevon Badloe, Seokwoo Kim, Chanwoong Park, Hojung Choi, Heon Lee, Junsuk Rho

**Affiliations:** 1 Department of Mechanical Engineering, Pohang University of Science and Technology (POSTECH), Pohang, 37673 Republic of Korea; 2 Department of Materials Science and Engineering, Korea University, Seoul, 02841 Republic of Korea; 3 Department of Chemical Engineering, Pohang University of Science and Technology (POSTECH), Pohang, 37673 Republic of Korea; 4 POSCO-POSTECH-RIST Convergence Research Center for Flat Optics and Metaphotonics, Pohang, 37673 Republic of Korea; 5 National Institute of Nanomaterials Technology (NINT), Pohang, 37673 Republic of Korea

**Keywords:** Metamaterials, Nanophotonics and plasmonics, Lithography, Displays

## Abstract

A single-step printable platform for ultraviolet (UV) metasurfaces is introduced to overcome both the scarcity of low-loss UV materials and manufacturing limitations of high cost and low throughput. By dispersing zirconium dioxide (ZrO_2_) nanoparticles in a UV-curable resin, ZrO_2_ nanoparticle-embedded-resin (nano-PER) is developed as a printable material which has a high refractive index and low extinction coefficient from near-UV to deep-UV. In ZrO_2_ nano-PER, the UV-curable resin enables direct pattern transfer and ZrO_2_ nanoparticles increase the refractive index of the composite while maintaining a large bandgap. With this concept, UV metasurfaces can be fabricated in a single step by nanoimprint lithography. As a proof of concept, UV metaholograms operating in near-UV and deep-UV are experimentally demonstrated with vivid and clear holographic images. The proposed method enables repeat and rapid manufacturing of UV metasurfaces, and thus will bring UV metasurfaces more close to real life.

## Abstract (web summary)

Facile nanostamping of the high-refractive index nanoparticle-embedded-resin for the direct fabrication of highly efficient metaholographic devices down to the deep-ultraviolet region.

## Graphical abstract

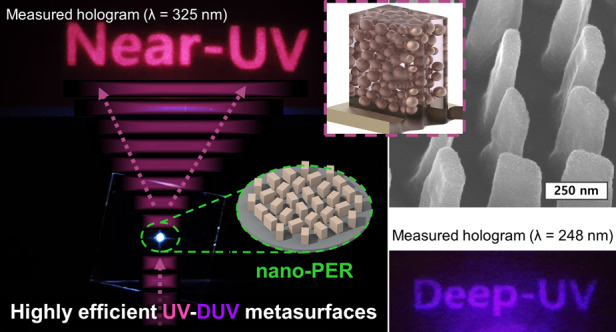

## Introduction

Ultraviolet (UV) optics play a critical role in numerous applications such as high-resolution imaging^1,2^, spectroscopy^3^, quantum optics^4,5^, photolithography^6^, and biosensing^7,8^. So far, UV light is mostly modulated using conventional bulky optical components which hinder the integration of compact systems. Moreover, conventional UV optics are limited in functionality, diversity, and manufacturability.

Metasurfaces composed of subwavelength structure arrays have been actively studied to replace conventional bulky optics, and with the exceptional ability to modulate light at the nanoscale have been applied to numerous applications such as metalenses^9,10^, biosensors^11,12^, metaholograms^13–21^, and color printing^22–26^. However, UV metasurfaces have long faced challenges such as a lack of UV transparent materials and high-resolution patterning techniques with low cost and high throughput. Conventional high-refractive-index materials used for metasurfaces usually have a narrow bandgap, resulting in high absorption of UV light^27^. So far, very few materials such as silicon nitride (SiN_x_)^28^, hafnium oxide (HfO_2_)^29^, zinc oxide (ZnO)^30^, and niobium pentoxide (Nb_2_O_5_)^31^ have been used for UV metasurfaces; however, the fabrication of those UV metasurfaces involves atomic layer deposition of thick layers or high-aspect-ratio etching, resulting in complicated fabrication process with the high cost and low throughput. Moreover, in all of the aforementioned UV metasurfaces, electron beam lithography (EBL) has been used for high-resolution patterning of subwavelength structures. These fabrication processes cause manufacturing limitations, such as high cost and low throughput, resulting in challenges in the commercialization of UV metasurfaces.

**Fig. 1:**
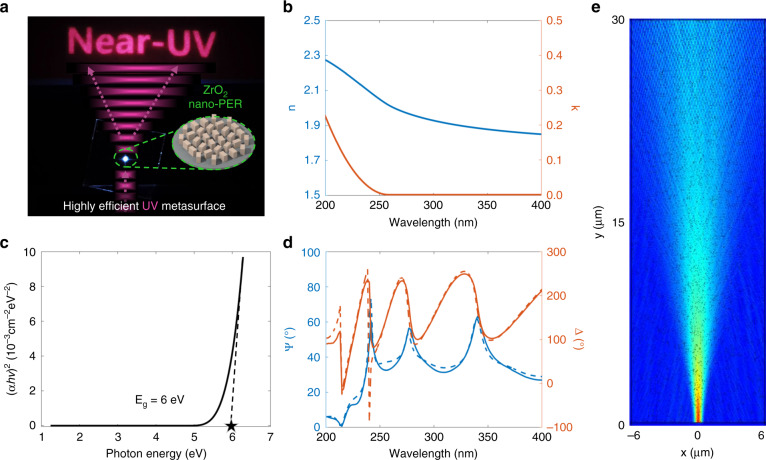
Optical characterization of ZrO_2_ nano-PER. **a** Schematic of the UV metasurface made up of ZrO_2_ nano-PER. **b** Measured complex refractive index of ZrO_2_ nano-PER film. Blue line represents the refractive index (*n*) and the orange line represents the extinction coefficient (*k*). **c** Calculated optical band gap of ZrO_2_ nano-PER film. **d** Measured amplitude ratio and phase difference of ZrO_2_ nano-PER film using ellipsometry. (Solid line: measured data; dashed line: model data) **e** Simulated scattering effect of a Gaussian beam propagating in the nano-PER film

Here, we introduce a one-step printable platform for high-efficiency metasurface operating over a broad UV range from near-UV to deep-UV region (Fig. 1a). Zirconium dioxide (ZrO_2_) nanoparticle embedded resin (nano-PER), a printable material with a large bandgap and high refractive index over a wide UV range, is newly proposed. ZrO_2_ nano-PER is synthesized by dispersing ZrO_2_ nanoparticles in a UV-curable resin. The proposed one-step printable platform enables direct replication of UV metasurfaces without the need for any secondary operations, resulting in extremely high throughput and low cost. The metasurface can achieve a high conversion efficiency owing to the high refractive index and low extinction coefficient of ZrO_2_ nano-PER. As a proof of concept, we experimentally demonstrate a metahologram operating in near-UV (325 nm) and deep-UV (248 nm).

## Results

### Characteristics of ZrO_2_ nano-PER

The key to a one-step printable UV metasurfaces is to produce a printable material that has a high refractive index (*n*) and low extinction coefficient (*k*) in the UV region. However, conventional printable materials such as imprint resin have a low refractive index of approximately 1.5. Recently, we developed a titanium dioxide (TiO_2_) nano-PER with *n* ≈ 1.95 in the visible region and silicon (Si) nano-PER with *n* ≈ 2.2 in the near-infrared region; however, both materials have severe absorption in the UV region owing to their small optical band gap^32–36^. Therefore, a high-*n* printable material with a large optical bandgap is required for high-efficiency UV metasurfaces.

The ZrO_2_ nano-PER developed here can be used as a UV transparent printable material with a high refractive index (Fig. 1b). The ZrO_2_ nano-PER has a large bandgap of 6 eV which leads to low absorption in the UV region (Fig. 1c, Fig. S1). The ZrO_2_ nano-PER is synthesized by dispersing 19 nm diameter ZrO_2_ nanoparticles with an 80% weight ratio into a UV-curable resin which makes the nano-PER printable (Fig. S2). As the weight ratio increases, the refractive index also increases (Fig. S3). However, the highest weight ratio is 80% because imprinting becomes difficult as the ratio increases over 80%. The complex refractive index of the ZrO_2_ nano-PER film is calculated by measuring the amplitude ratio (Ψ) and phase difference (Δ) between the *s* and *p* components of three different angles (65˚, 70˚, 75˚) using ellipsometry (Fig. S4). In order for the nano-PER to operate as a meta-atom, the nano-PER should act as a homogeneous effective medium. The measured Ψ and Δ fit well with the Tauc-Lorentz model^37^, which provides the validity of the ZrO_2_ nano-PER as the homogeneous effective medium (Fig. 1d). Moreover, the scattering effect of a Gaussian beam in the ZrO_2_ nano-PER is simulated to confirm that the nano-PER acts as an effective medium (Fig. 1e). The diameter of the ZrO_2_ nanoparticles follows a Gaussian distribution of 19 nm on average. Owing to small particle size, the scattering effect is negligible, and the Gaussian beam maintains its shape as it propagates. These results prove that the ZrO_2_ nano-PER can act as an effective medium and can be applied for use in a metasurface.

### Design of high-efficiency UV meta-atoms

**Fig. 2:**
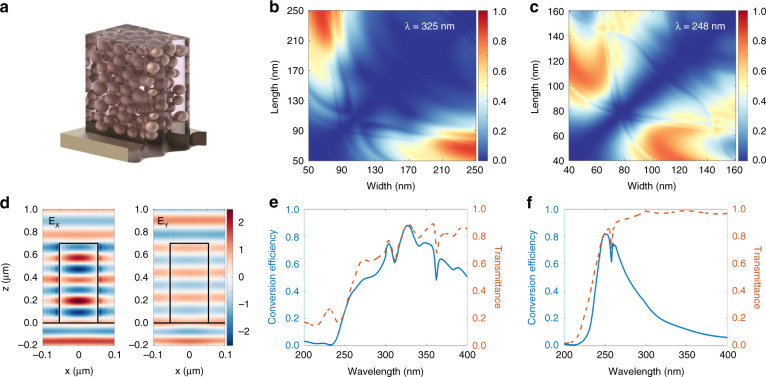
Design and simulation of the high-efficiency meta-atom operating in near-UV and deep-UV. **a** Schematic illustration of the printable meta-atom consisting of ZrO_2_ nano-PER. **b** Simulated conversion efficiencies for meta-atoms at λ = 325 nm. Width and length parameters are varied from 50 nm to 250 nm with fixed height (700 nm) and period (300 nm). **c** Simulated conversion efficiencies for meta-atoms at λ = 248 nm. Width and length parameters are varied from 40 nm to 160 nm with fixed height (700 nm) and period (200 nm). **d** Simulated electric field profiles at λ = 248 nm. Real part of the *x* component (left) and *y* component (right) of the electric field. Simulated conversion efficiency and transmittance of designed meta-atom operating in **e** λ = 325 nm and **f** λ = 248 nm

Rigorous coupled-wave analysis (RCWA)^38^ was used to simulate the transmission properties of meta-atoms consisting of ZrO_2_ nano-PER. To achieve full phase modulation with broadband property, the concept of the Pancharatnam-Berry phase (PB phase)^16^, also known as geometric phase^39,40^, is used to physically realize the required phase profile (Supplementary Note 1). PB phase uses an anisotropic meta-atom which is a birefringence (Fig. 2a). Transmitted light with a converted handedness of polarization (cross-polarization) has a phase delay of 2*θ*. The amplitude of the cross-polarized component is defined as a conversion efficiency that is directly related to the efficiency of the meta-atom.

The final goal of this work is to design high-efficiency meta-atoms operating from near-UV (325 nm) down to deep-UV (248 nm). For the near-UV meta-atom, we calculate the conversion efficiencies of meta-atoms with varying lengths and widths from 50 nm to 250 nm with a fixed height of 700 nm and periodicity of 300 nm (Fig. 2b). The meta-atom with a length of 250 nm and a width of 65 nm has a conversion efficiency of 88% at a wavelength of 325 nm. For deep-UV meta-atom, we calculate conversion efficiencies of meta-atom varying lengths and widths from 40 nm to 160 nm with a fixed height of 700 nm and periodicity of 200 nm (Fig. 2c). The meta-atom with a length of 110 nm and a width of 45 nm has a conversion efficiency of 81% at a wavelength of 248 nm. The height is optimized for maximum conversion efficiency (Fig. S5), and periodicity is determined to be smaller than the operating wavelength to suppress the diffraction of transmitted light. Notably, candidate meta-atoms near the target geometry still have high efficiency, therefore some fabrication errors are acceptable. The ideal PB phase meta-atom should provide a π–phase difference between the *x* and *y* components of the electric field (**E**_**x**_ and **E**_**y**_), and act as a half-wave plate. We plot real values of the propagating electric field profiles of *x*- and *y*- polarized light in designed meta-atoms at the designed wavelength of 325 nm and 248 nm (Fig. 2d, Fig. S6). We confirm that designed meta-atoms provide a π–phase difference between the *x* and *y* components of the electric field and act as a half-wave plate, therefore operating as an ideal PB phase meta-atom. Owing to the broadband property of the PB phase, the meta-atom designed for 325 and 248 nm has high efficiency and low zero-order efficiency near the target wavelength, respectively (Fig. 2e, f, Fig. S7). Owing to the low extinction coefficient of ZrO_2_ nano-PER, the designed meta-atom has high transmittance and low absorption in the UV region (Fig. S8).

### One-step printable platform for ZrO_2_ nano-PER based UV metasurface

**Fig. 3:**
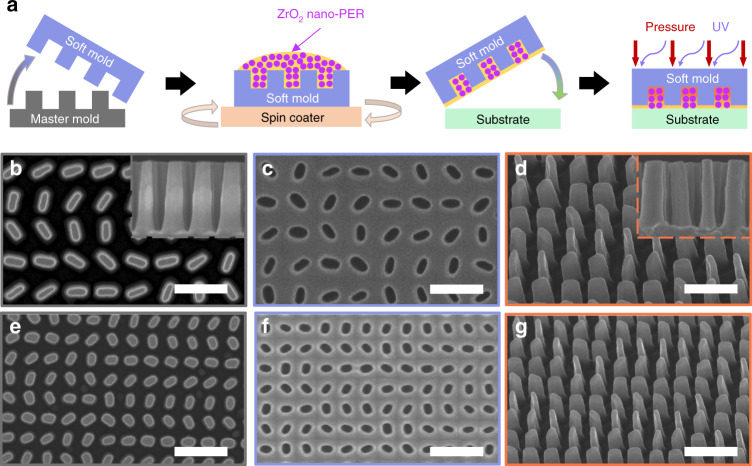
Fabrication of UV metaholographic device using the one-step printable process of ZrO2 nano-PER. **a** Schematic of the one-step printable platform for fabrication of the UV metaholographic device. SEM images of **b** the master mold, **c** the soft mold, and **d** the one-step printed UV metasurface of the target hologram designed for λ = 325 nm. (Inset) cross-section view. SEM images of **e** the master mold, **f** the soft mold, and **g** the one-step printed UV metasurface of the target hologram designed for λ = 248 nm. All scale bars: 500 nm

A schematic for the one-step printable platform for ZrO_2_ nano-PER-based UV metasurface is described in Fig. 3a. First, master molds with different scales for metasurfaces operating at λ = 325 nm and 248 nm are fabricated by conventional EBL, mask deposition, and lift-off process, respectively (details in the *Methods*, Fig. 3b, e). Then, the fabricated master molds are covered with a hard-polydimethylsiloxane (h-PDMS)/PDMS bilayer and cured by heat for the successful transfer of extremely small meta-atoms with 50-nm resolution (Fig. 3c, f)^41^. The 80 wt% ZrO_2_ nano-PER in the MIBK solvent is spin-coated on soft molds to achieve the uniform ZrO_2_ nano-PER thin film with an optimal residual layer thickness which affects the final conversion efficiency and reflectivity of UV metasurfaces(Fig. 3d, g)^32^. It is beyond any doubt that the conformally coated ZrO_2_ nano-PER layer fits perfectly with the flat target substrate. Plus, an additional PMMA layer between the ZrO_2_ nano-PER and substrates can underpin non-trivial enhancement of work of adhesion, which is suitable for the facile transfer on any arbitrary substrate^32^. To finish, the adequate pressurization and UV illumination achieve UV metasurfaces operating at λ = 325 nm and 248 nm, respectively.

### Design and demonstration of a UV metahologram

**Fig. 4:**
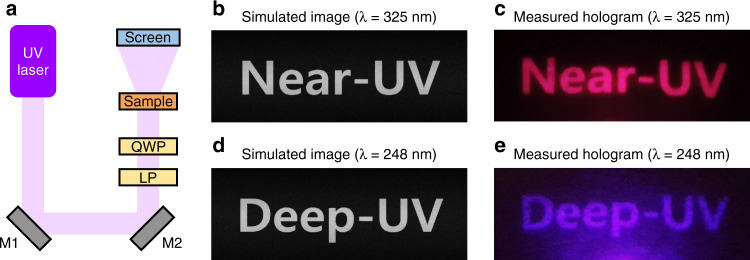
Demonstration of the UV metahologram. **a** Optical setup for ZrO_2_ nano-PER based UV metahologram (UV laser: HeCd laser for 325 nm, KrF laser for 248 nm; LP: linear polarizer; QWP: quarter-wave plate; Screen: UV sensor card). **b** Simulated and **c** measured image of the target hologram designed for λ = 325 nm. **d** Simulated and **e** measured image of the target hologram designed for λ = 248 nm

We design a simple Fraunhofer hologram as a representative wavefront shaping function of the designed UV metasurface. The Gerchberg-Saxton (GS) algorithm is used to retrieve the phase map for high-quality phase-only holograms^42^. Since Fraunhofer approximation results in a pincushion-like distortion in the recovered hologram, barrel distortion is used to compensate for the distortion by trial and error. By modulating the phase profile with the designed meta-atoms, we design and demonstrate high-quality UV metaholograms operating in the near-UV and deep-UV. The optical setup for UV metaholograms is prepared as shown in Fig. 4a. A helium cadmium (HeCd) laser is used for λ = 325 nm, and a krypton fluoride (KrF) laser is used for λ = 248 nm. Two UV wave plates, a linear polarizer, and a quarter-wave plate are used to create the circularly polarized input beam. A UV sensor card is used to visualize the UV hologram. As we expected, demonstrated holographic images match well with simulated images and show a vivid and clear image in the near-UV (Fig. 4b, c) and deep-UV (Fig. 4d, e). Moreover, we experimentally measure the conversion efficiency of both metaholograms. The metahologram designed for near-UV regime has a measured conversion efficiency of 72.3% at λ = 325 nm, and the metahologram designed for deep-UV regime has a measured conversion efficiency of 48.6% at λ = 248 nm (**Table Sl**). We also confirmed that this work has higher efficiency compared to previously reported UV metasurfaces (Table S2).

## Discussions

In summary, we proposed and verified a one-step printable platform in which high-efficiency metasurfaces operating from near-UV to deep-UV can be replicated repeatably with low cost and high throughput. In detail, single ZrO_2_ nano-PER metasurface can be fabricated in 15 minutes and costs around 1.39 USD (Table S3). The ZrO_2_ nano-PER is synthesized as a printable material having high UV transparency and refractive index by dispersing ZrO_2_ nanoparticles in a UV-curable resin. Owing to the UV-curable matrix, the UV metasurface consisting of the ZrO_2_ nano-PER can be fabricated with one step of nanoimprint lithography without any secondary operations such as etching and deposition. The refractive index of the ZrO_2_ nano-PER is high enough to confine the light well and the extinction coefficient is low enough to minimize the absorption, resulting in high conversion efficiency. The simulated conversion efficiency of the designed meta-atoms achieve 88% for λ = 325 nm and 81% for λ = 248 nm, respectively. As a proof of concept, we experimentally demonstrate a clear and vivid metahologram operating in near-UV and deep-UV. The demonstrated hologram has a conversion efficiency of 72.3% for λ = 325 nm and 48.6% for λ = 248 nm, respectively. We believe that this work will be a decisive improvement in the practicality of UV metasurfaces.

## Methods

### Synthesis of ZrO_2_ nano-PER

The ZrO_2_ nano-PER was prepared by mixing ZrO_2_ NPs dispersed in MIBK (DT-ZROSOL-30MIBK (N10), Ditto technology), monomer (dipentaerythritol penta-/hexa- acrylate, Sigma-Aldrich), photo-initiator (1-Hydroxycyclohexyl phenyl ketone, Sigma-Aldrich), and MIBK solvent (MIBK, Duksan general science). The mixing ratio was controlled to achieve a weight ratio of 4 wt % for ZrO_2_ NPs, 0.7 wt % for monomer, and 0.3 wt % for photo-initiator.

### Fabrication of the master mold

A Si substrate was used for the master mold. The meta-atoms were transferred onto a bilayer of two positive tone photoresists (495 PMMA A6, MicroChem & 950 PMMA A2, MicroChem) using the standard EBL process (ELIONIX, ELS-7800; acceleration voltage: 80 kV, beam current: 100 pA). The exposed patterns were developed by MIBK/IPA 1:3 developer mixed solution. An 80 nm-thick chromium (Cr) layer was deposited using electron beam evaporation (KVT, KVE-ENS4004). The lifted-off Cr meta-atoms were used as an etching mask for the Si substrate. Cr patterns were transferred onto the Si substrate using a dry etching process (DMS, silicon/metal hybrid etcher). The remaining Cr etching mask was removed by Cr etchant (CR-7).

### Fabrication of the soft mold

*h*-PDMS was prepared by mixing 3.4 g of vinylmethyl copolymers (VDT-731, Gelest), 18 μL of platinum-caralyst (SIP6831.2, Gelest), 0.1 g of the modulator (2,4,6,8- tetramethyl-2,4,6,8-tetravinylcyclotetrasiloxane, Sigma-aldrich), 2 g of toluene, and 1 g of siloxane-based silane reducing agent (HMS-301, Gelest). The *h*-PDMS was spin-coated on the master mold at 1,000 rpm for 60 s, then baked at 70 °C for 2 h. A mixture of a 10:1 weight ratio of PDMS (Sylgard 184 A, Dow corning) and its curing agent (Sylgard 184 B, Dow corning) was poured on the *h*-PDMS layer and cured at 80 °C for 2 h. The cured soft mold was detached from the master mold, then used to replicate the nano-PER structure.

### Pre-treatment of the soft mold

Fluorosurfactant ((tridecafluoro-1,1,2,2-tetrahydrooctyl) trichlorosilane) is coated on the soft mold by vaporized coating at 130 °C for 5 min to decrease the surface tension of the soft mold.

## Supplementary information

Supplementary Information

## Data Availability

The data that support the findings of this study are available from the corresponding author upon reasonable request.
