# Supplementary material for: One-step printable platform for high-efficiency metasurfaces down to the deep-ultraviolet region
Source: Light Sci Appl. 2023 Mar 8;12:68. doi: 10.1038/s41377-023-01086-6 (PMC9992517; doi:10.1038/s41377-023-01086-6)
Supplement: Supplementary file 1 — Supplementary Information [file 41377_2023_1086_MOESM1_ESM.pdf]

Supplementary Information for

One-step printable platform for high-efficiency metasurfaces down to the deep ultraviolet region

Joohoon Kim,[1,†] Wonjoong Kim,[2,†] Dong Kyo Oh,[1,†] Hyunjung Kang,[1] Hongyoon Kim,[1] Trevon Badloe,[1] Seokwoo Kim,[1] Chanwoong Park,[2] Hojung Choi,[2] Heon Lee,[2,*] and Junsuk Rho[1,3,4,5,*]

[1]Department of Mechanical Engineering, Pohang University of Science and Technology (POSTECH), Pohang 37673, Republic of Korea

[2]Department of Materials Science and Engineering, Korea University, Seoul, 02841, Republic of Korea.

[3]Department of Chemical Engineering, Pohang University of Science and Technology (POSTECH), Pohang 37673, Republic of Korea

[4]POSCO-POSTECH-RIST Convergence Research Center for Flat Optics and Metaphotonics, Pohang 37673, Republic of Korea

[5]National Institute of Nanomaterials Technology (NINT), Pohang 37673, Republic of Korea

[†]These authors contributed equally to this work.

*Corresponding author. E-mail: jsrho@postech.ac.kr; heonlee@korea.ac.kr

Supplementary Note 1. Jones matrix analysis of rotated anisotropic meta-atom.

In this work, an anisotropic meta-atom is used to achieve the geometric phase. The anisotropic meta-atom can be expressed with a Jones matrix **J**, given by

$$\mathbf{J} = \begin{bmatrix} t_{xx} & 0 \\ 0 & t_{yy} \end{bmatrix}, \quad \text{(S1)}$$

where t_l and t_s represent the complex transmission coefficients for light polarized along the long and short axes of the meta-atoms, respectively. The Jones matrix **T** for θ rotated meta-atoms can be expressed using a rotational matrix $\mathbf{R}(\theta)$, as follows

$$\mathbf{T} = \mathbf{R}(-\theta)\ \mathbf{J}\ \mathbf{R}(\theta). \quad \text{(S2)}$$

Finally, the transmitted electric field E_T can be calculated using **T**, as follows

$$E_T = \frac{t_l + t_s}{2}\begin{bmatrix} 1 \\ \mp i \end{bmatrix} + \frac{t_l - t_s}{2} e^{\pm i2\theta} \begin{bmatrix} 1 \\ \pm i \end{bmatrix}, \quad \text{(S3)}$$

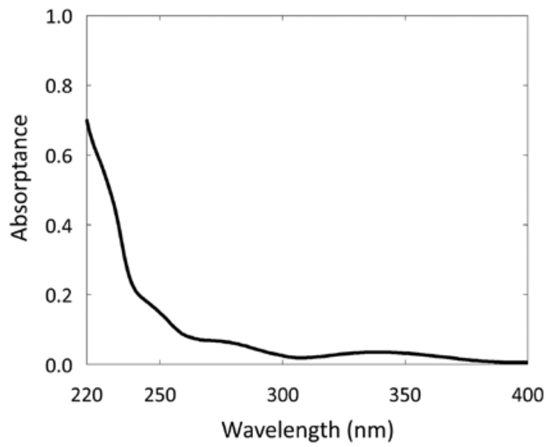

Figure S1. Measured absorption spectrum of zirconium dioxide (ZrO_2) nanoparticle-embedded-resin (nano-PER) film.

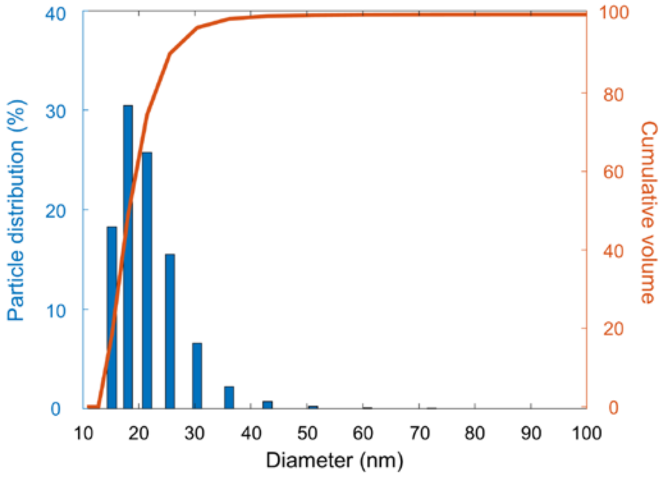

Figure S2. Measured particle size distribution of ZrO_2 nano-PER using laser diffraction analyzer.

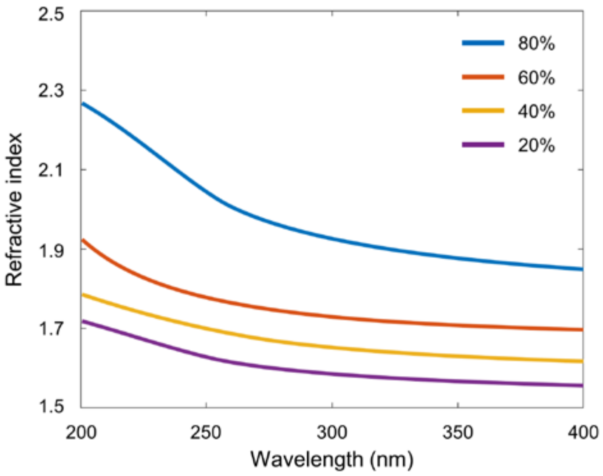

Figure S3. Measured refractive index of ZrO_2 nano-PER with different ZrO_2 weight ratio.

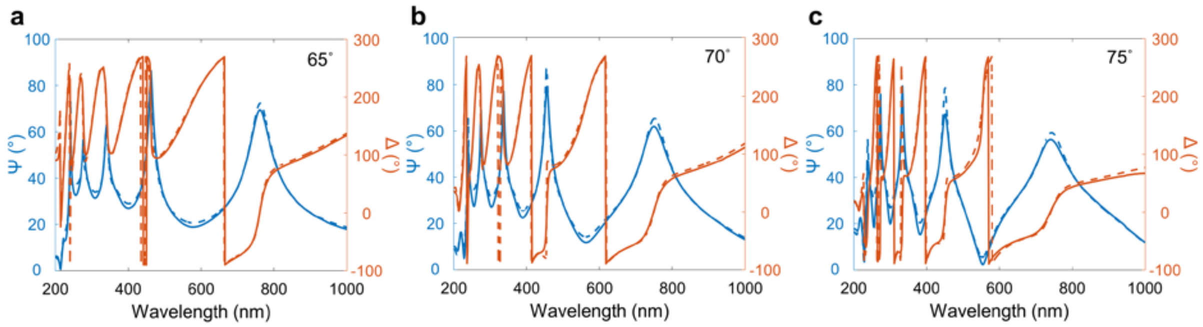

Figure S4. Measured amplitude ratio and phase difference of ZrO_2 nano-PER film with three different incident angles: **(a)** 65˚, **(b)** 70˚, **(c)** 75˚.

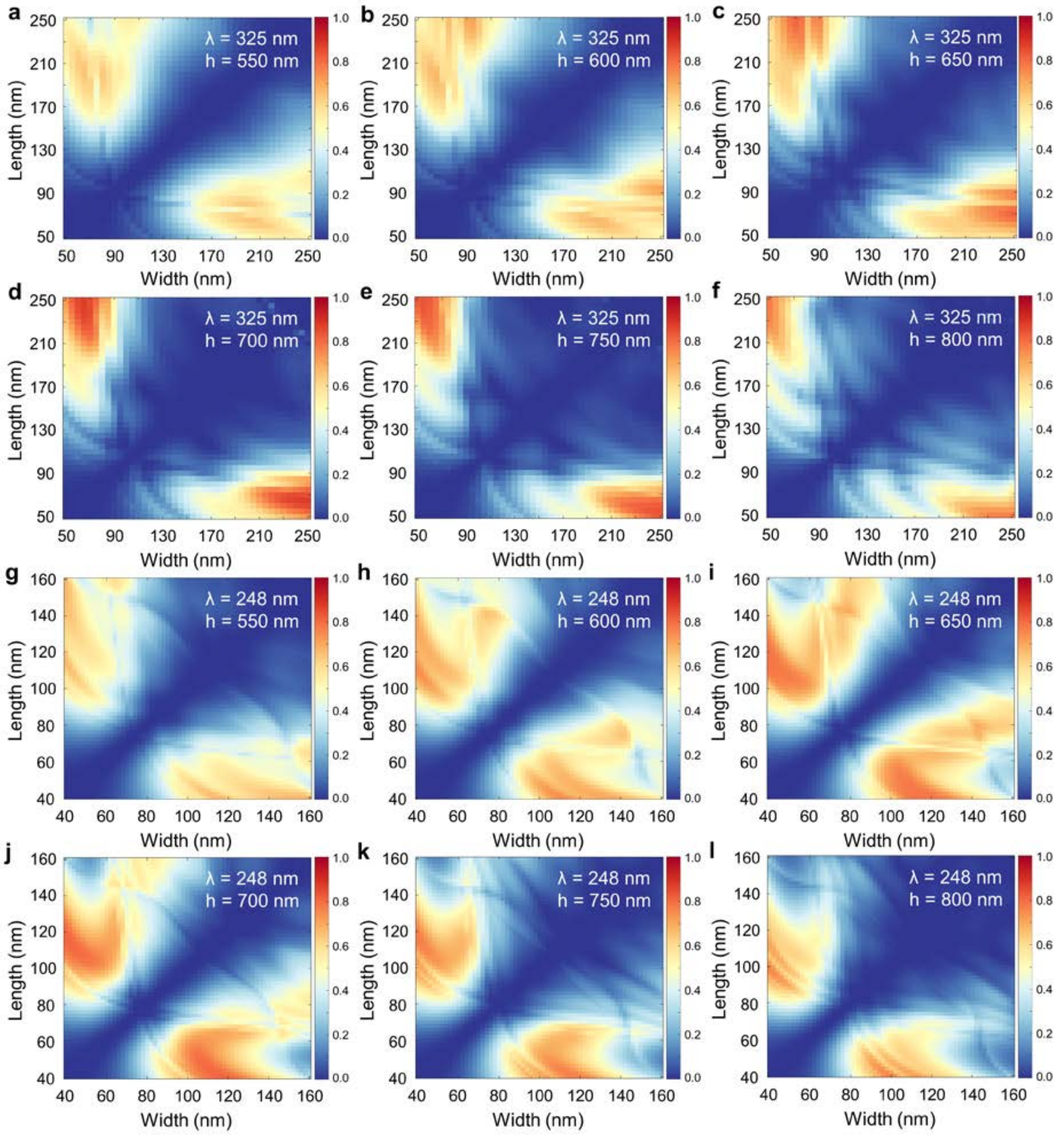

Figure S5. Simulated conversion efficiencies varying the height from 550 nm to 800 nm at **(a-f)** λ = 325 nm, and **(g-l)** λ = 248 nm.

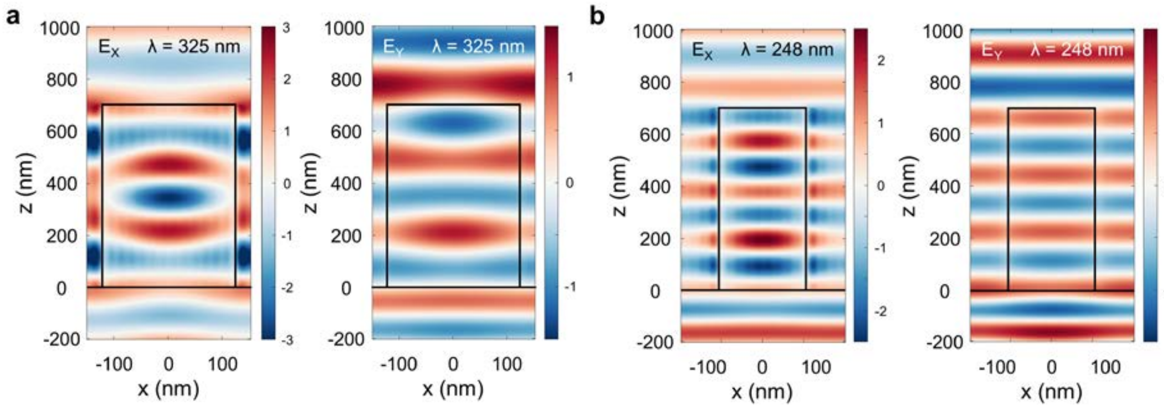

Figure S6. Real part of the simulated electric field profiles at **(a)** λ = 325 nm, and **(b)** λ = 248 nm. Real part of the x-component (left) and y-component (right) of the electric field.

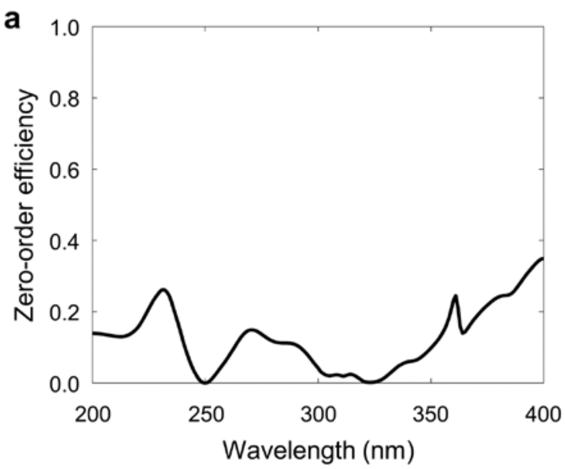

Figure S7. Simulated zero-order efficiency of the designed meta-atom for **(a)** λ = 325 nm and **(b)** λ = 248 nm.

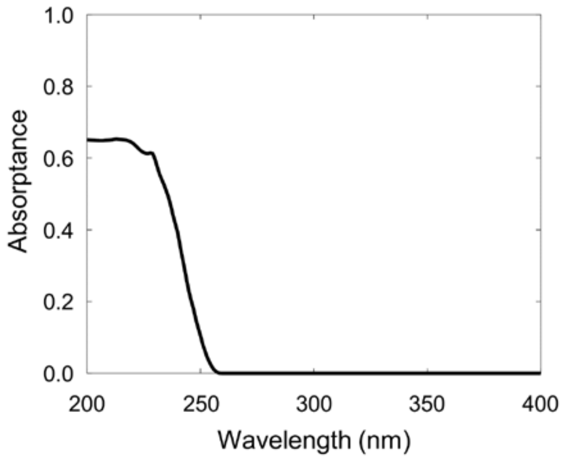

Figure S8. Simulated absorptance of the designed meta-atom for λ = 325 nm.

Table S1. Calculation of the measured conversion efficiency.

Wavelength (nm)	Reference (μW)	Converted beam (μW)	Conversion efficiency (%)
325	45.87	33.16	72.3
248	83.63	40.64	48.6

Table S2. Efficiency comparison of this work and previously reported UV metasurfaces.

Reference	Material	Wavelength (nm)	Theoretical efficiency (%)	Experimental efficiency (%)
This work	ZrO_2 nano-PER	325	88	72
		248	81	49
[1]	Si	380	-	15
		290	-	10
[2]	ZnO	197	-	7.7
[3]	Nb_2O_5	355	83	80
[4]	HfO_2	364	-	55
		266	-	56
[5]	SiN	326	72	-

Table S3. Breakdown of the cost for ZrO_2 nano-PER fabrication.

**Material**	Dipentaerythritol penta-/hexa-acrylate	1-Hydroxycyclohexyl phenyl ketone	Methyl Isobutyl Ketone (MIBK) (99.5%)	DT-ZROSOL-30MIBK (N10)
**Role**	Monomer	Photo-initiator	Solvent	30wt% ZrO_2 NP-dispersed MIBK
**Supplier**	Sigma-Aldrich	Sigma-Aldrich	Duksan general science	Ditto technology
**Volume (or weight)**	100 mL	50 g	1 L	200 g
**Price (USD)**	80	70	9	215
**Volume (or weight) per sample**	0.1 mL	0.85 g	0.7 mL	0.104 g
**Price per sample (USD)**	0.08	1.19	0.0063	0.1118

References

1 Deng, Y. *et al.* All-silicon broadband ultraviolet metasurfaces. *Adv. Mater.* **30**, 1802632 (2018).

2 Tseng, M. L. *et al.* Vacuum ultraviolet nonlinear metalens. *Sci. Adv.* **8**, eabn5644 (2022).

3 Huang, K. *et al.* Ultraviolet metasurfaces of ≈80% efficiency with antiferromagnetic resonances for optical vectorial anti-counterfeiting. *Laser and Photonics Rev.* **13**, 1800289 (2019).

4 Zhang, C. *et al.* Low-loss metasurface optics down to the deep ultraviolet region. *Light Sci. Appl.* **9**, 55 (2020).

5 Kim, J. *et al.* Photonic encryption platform via dual-band vectorial metaholograms in the ultraviolet and visible. *ACS Nano* **16**, 3546-3553 (2022).
